# Supplementary material for: Bacillus megaterium HgT21: a Promising Metal Multiresistant Plant Growth-Promoting Bacteria for Soil Biorestoration
Source: Microbiol Spectr. 2022 Aug 18;10(5):e00656-22. doi: 10.1128/spectrum.00656-22 (PMC9604106; doi:10.1128/spectrum.00656-22)
Supplement: Supplemental file 1 — Supplemental material. Download spectrum.00656-22-s0001.pdf, PDF file, 0.7 MB [file spectrum.00656-22-s0001.pdf]

Supplemental Figures

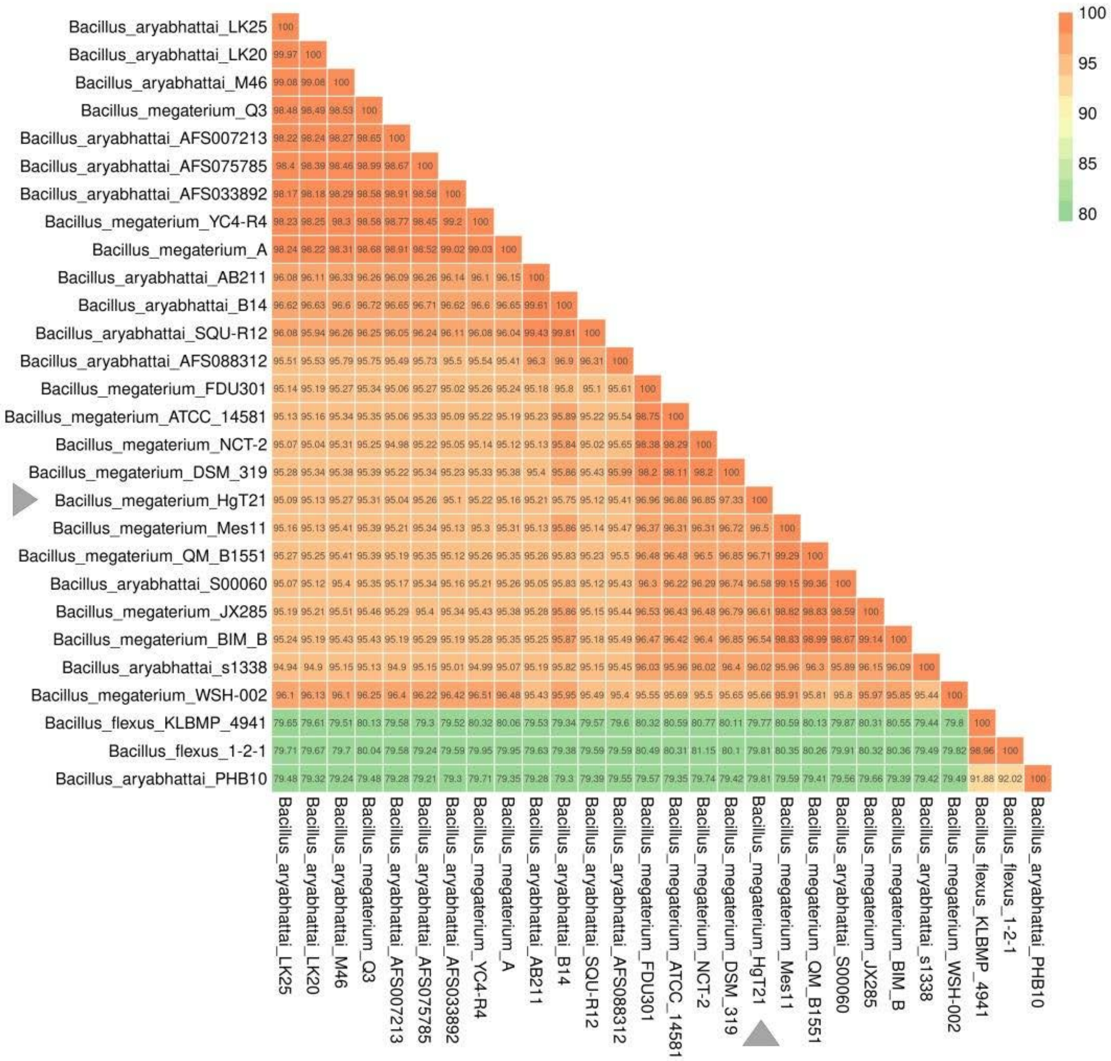

Fig. S1. Heatmap of pairwise average nucleotide identity (ANI) values for 28 whole genome sequenced species closely related to HgT21 strain (including in the analysis). Cool colors represent poor similarity between species, while warm colors depict pairwise highly similar. ANI values are included in each cell of the heatmap.

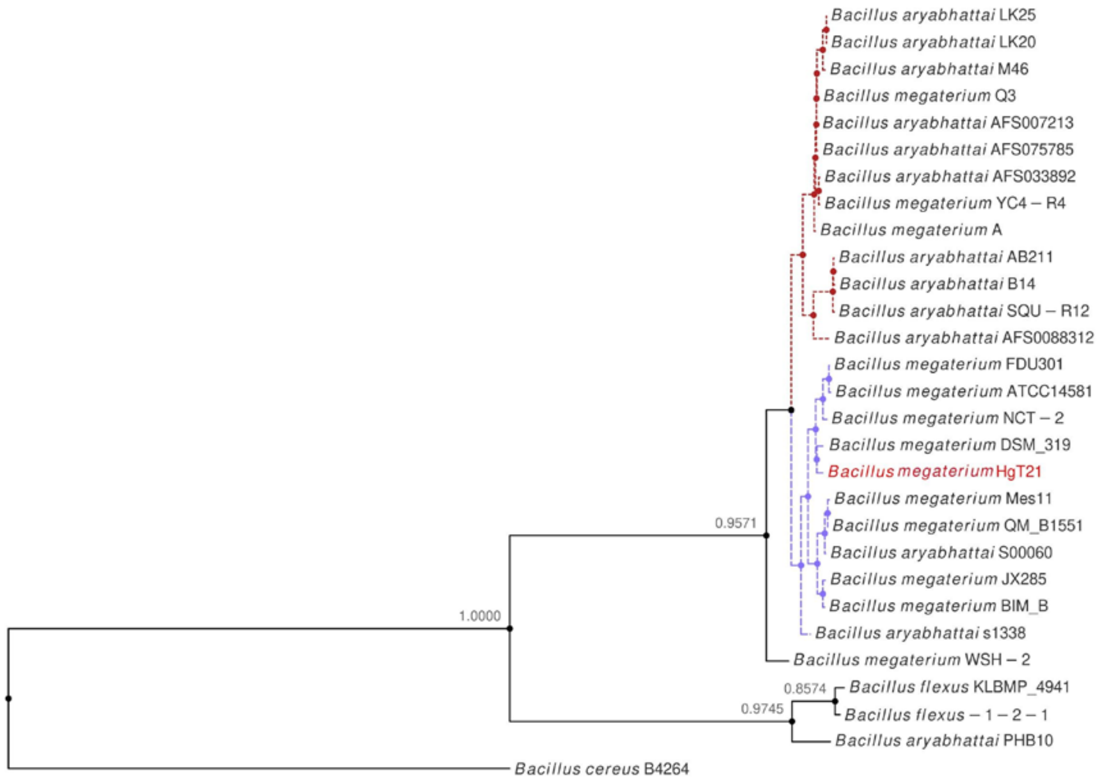

Fig. S2. Phylogenetic relationship of *Bacillus megaterium* HgT21 based on 8,098 orthogroups. Only values larger than 0.5 are shown. The dotted lines represent the two main clades of *B. megaterium* - *B. aryabhattai* identified in the analysis.

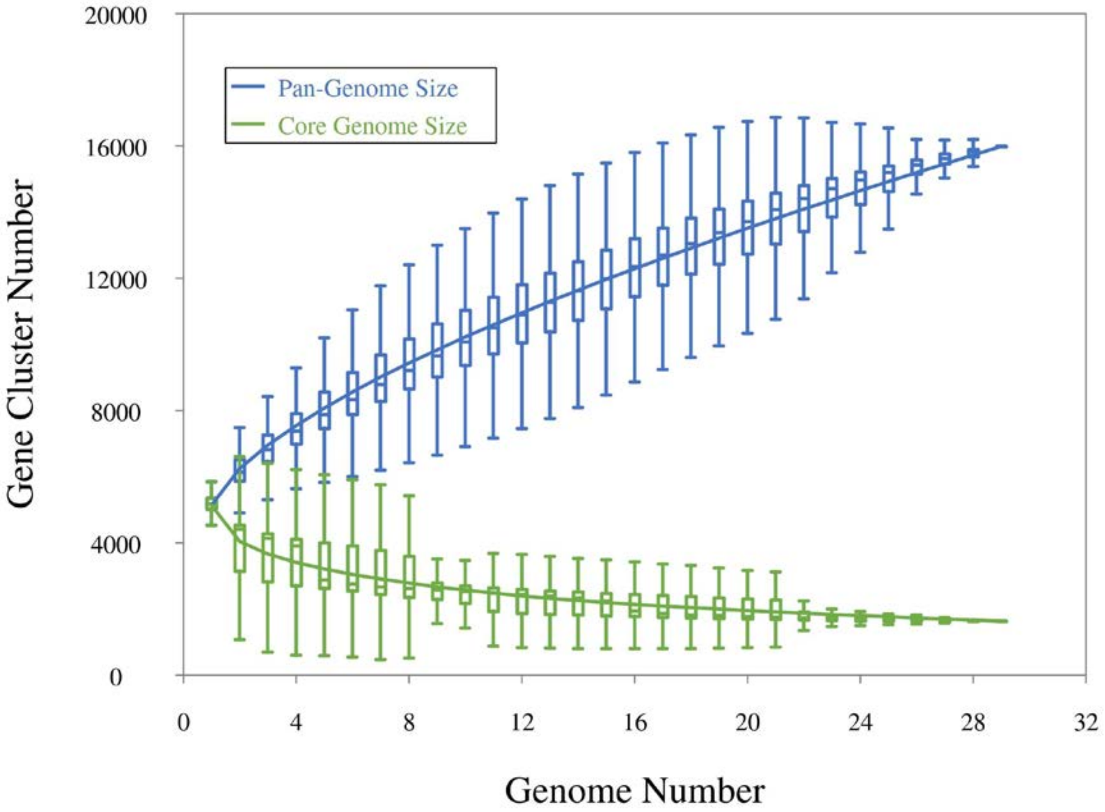

Fig. S3. Gene accumulation curves of the species closely related to HgT21. Pan- (blue) and core-genome (green). The estimation was made by including genomes one by one**.**

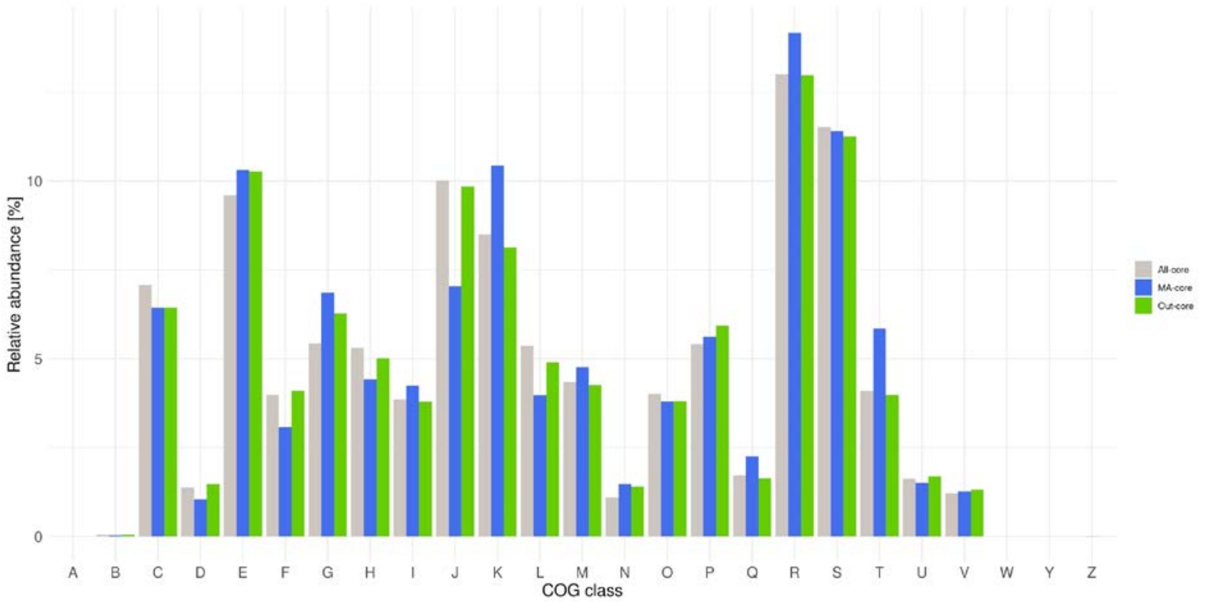

Fig. S4. Comparison of clusters of orthologous groups (COGs) in gene cores shaped by the megaterium-aryabhattai clade (MA; blue), outgroup clade (Out; green) and all species (All; gray). [A] RNA processing and modification [B] Chromatin structure and dynamics [C] Energy production and conversion [D] Cell cycle control, cell division, chromosome partitioning [E] Amino acid transport and metabolism [F] Nucleotide transport and metabolism [G] Carbohydrate transport and metabolism [H] Coenzyme transport and metabolism [I] Lipid transport and metabolism [J] Translation, ribosomal structure and biogenesis [K] Transcription [L] Replication, recombination and repair [M] Cell wall/membrane/envelope biogenesis [N] Cell motility [O] Post-translational modification, protein turnover, and chaperones [P] Inorganic ion transport and metabolism [Q] Secondary metabolites biosynthesis, transport, and catabolism [R] General function prediction only [S] Function unknown [T] Signal transduction mechanisms [U] Intracellular trafficking, secretion, and vesicular transport [Z] Cytoskeleton.

Supplemental Tables

Table S1. Assembly accession and number of protein-coding sequences of the genomes used for HgT21 phylogenetic analysis.

Species	Assembly accession	#protein-coding sequences
Bacillus aryabhattai AB211	GCA_001858395.1	5,323
Bacillus aryabhattai B14	GCA_002167185.1	4,673
Bacillus aryabhattai PHB10	GCA_002265635.1	4,165
Bacillus flexus 1-2-1	GCF_005577315.1	4,041
Bacillus megaterium FDU301	GCA_013146705.1	6,928
Bacillus megaterium Q3	GCA_001050455.1	5,264
Bacillus aryabhattai AFS007213	GCA_002555815.1	5,404
Bacillus aryabhattai S00060	GCA_014138775.1	6,358
Bacillus flexus KLBMP 4941	GCF_002024265.1	4,156
Bacillus megaterium JX285	GCA_002009195.1	5,604
Bacillus megaterium QM B1551	GCA_000025825.1	5,629
Bacillus aryabhattai AFS033892	GCA_002588685.1	5,575
Bacillus aryabhattai LK20	GCA_001043825.1	5,396
Bacillus aryabhattai s1338	GCA_014932885.1	6,287
Bacillus megaterium A	GCA_009497655.1	5,257
Bacillus megaterium Mes11	GCA_013458535.1	6,194
Bacillus megaterium WSH-002	GCA_000225265.1	5,274
Bacillus aryabhattai AFS075785	GCA_002569785.1	5,169
Bacillus aryabhattai LK25	GCA_001038965.1	5,326
Bacillus aryabhattai SQU-R12	GCA_002208605.1	5,635
Bacillus megaterium BIM B-1314D	GCA_013389435.1	5,978

Bacillus megaterium NBRC 15308	GCA_006094495.1	5,741
Bacillus megaterium YC4-R4	GCA_003072605.2	5,370
Bacillus aryabhattai AFS088312	GCA_002569015.1	5,509
Bacillus aryabhattai M46	GCA_001619595.1	4,915
Bacillus cereus B4264	GCF_000021205.1	5,398
Bacillus megaterium DSM 319	GCA_000025805.1	5,124
Bacillus megaterium NCT-2	GCA_000334875.3	5,835
Bacillus megaterium HgT21		5,771
**Total**		157,299

*****This study

Table S2. Strain specific genes identified in *B. aryabhattai* HgT21.

ID	Contig	Start	End	Description	Pfam	COG_ID	COG_Description
101	11	24406	24522	hypothetical protein	-	-	-
109	11	31485	31309	hypothetical protein	-	-	-
110	11	32098	31889	hypothetical protein	-	-	-
113	11	35837	36031	hypothetical protein	-	-	-
114	11	36112	36657	hypothetical protein	Putative inner membrane protein (DUF1819)	-	-
115	11	36669	37235	putative cytoplasmic protein	Domain of unknown function (DUF1788)	-	-
116	11	37253	40831	ATPase-like protein	-	-	-
117	11	40847	44341	putative type II restriction enzyme methylase subunit	Eco57I restriction-modification methylase	COG1002	Type II restriction enzyme, methylase subunits
118	11	44417	46975	putative cytoplasmic protein	PglZ domain	-	-
119	11	46993	49050	putative ATP-dependent protease	Lon protease (S16) C-terminal proteolytic domain	COG4930	Predicted ATP-dependent Lon-type protease
119	11	46993	49050	putative ATP-dependent protease	Putative ATP-dependent Lon protease	COG4930	Predicted ATP-dependent Lon-type protease
11	10	17282	16839	Rrf2 family transcriptional regulator, group III	Transcriptional regulator	COG1959	Predicted transcriptional regulator
120	11	51057	49639	hypothetical protein	Reverse transcriptase (RNA-dependent DNA polymerase)	-	-
121	11	51747	51076	hypothetical protein	SMODS and SLOG-associating 2TM effector domain family 5	-	-
122	11	52230	52090	hypothetical protein	-	-	-
1237	1	821033	821176	hypothetical protein	-	-	-
1303	1	883805	883966	hypothetical protein	-	-	-
1315	1	892858	892736	hypothetical protein	-	-	-
1441	1	1014668	1014862	hypothetical protein	-	-	-

1482	1	1048756	1048619	hypothetical protein	-	-	-
1511	1	1071920	1072090	Glutathione-regulated potassium-efflux system ancillary protein KefG	-	-	-
1512	1	1072513	1072346	hypothetical protein	-	-	-
1513	1	1072619	1072488	hypothetical protein	-	-	-
1518	1	1075925	1076257	Ankyrin	Ankyrin repeats (3 copies)	Arp	FOG: Ankyrin repeat
1523	1	1078768	1078914	hypothetical protein	-	-	-
1529	1	1082561	1082677	hypothetical protein	-	-	-
1535	1	1090469	1090353	hypothetical protein	-	-	-
1548	1	1096405	1096578	hypothetical protein	-	-	-
1550	1	1097100	1096921	hypothetical protein	-	-	-
1580	1	1127918	1128358	hypothetical protein	-	-	-
1585	1	1133563	1134132	hypothetical protein	Bacterial regulatory proteins, tetR family	AcrR	Transcriptional regulator
1586	1	1134436	1135557	Heme efflux system permease HrtB	FtsX-like permease family	LolE	ABC-type transport system, involved in lipoprotein release, permease component
1586	1	1134436	1135557	Heme efflux system permease HrtB	MacB-like periplasmic core domain	LolE	ABC-type transport system, involved in lipoprotein release, permease component
1587	1	1135558	1136244	Heme efflux system ATPase HrtA	ABC transporter	SalX	ABC-type antimicrobial peptide transport system, ATPase component
1589	1	1137166	1137303	hypothetical protein	-	-	-
1599	1	1145701	1145853	hypothetical protein	-	-	-
1602	1	1148798	1148923	hypothetical protein	-	-	-
1613	1	1159464	1159315	hypothetical protein	-	-	-
1614	1	1159579	1159737	hypothetical protein	Cytochrome P450	CypX	Cytochrome P450
1635	1	1178417	1178563	hypothetical protein	-	-	-
1638	1	1180570	1180686	hypothetical protein	-	MhpC	Predicted hydrolases or acyltransferases (alpha/beta hydrolase superfamily)
1651	1	1193261	1193118	hypothetical protein	-	-	-

1653	1	1195714	1195929	hypothetical protein	Protein of unknown function (DUF3243)	-	-
1658	1	1200150	1200347	hypothetical protein	-	-	-
1659	1	1200381	1200497	hypothetical protein	-	-	-
1664	1	1204104	1204724	Flavohemoprotein (Hemoglobin-like protein) (Flavohemoglobin) (Nitric oxide dioxygenase) (EC 1.14.12.17)	NAD(P)H-binding	COG2910	Putative NADH-flavin reductase
1672	1	1213027	1213167	hypothetical protein	-	-	-
1691	1	1230864	1231202	hypothetical protein	-	-	-
1693	1	1232413	1232580	RNA polymerase sigma factor SigZ	-	-	-
1694	1	1232713	1232925	RNA polymerase sigma factor SigZ	Sigma-70, region 4	RpoE	DNA-directed RNA polymerase specialized sigma subunit, sigma24 homolog
1695	1	1232945	1233250	hypothetical protein	-	-	-
1696	1	1233295	1234107	hypothetical protein	Family of unknown function (DUF5643)	-	-
1703	1	1238307	1238444	hypothetical protein	-	-	-
1717	1	1252083	1252271	hypothetical protein	-	-	-
1723	1	1258655	1258789	hypothetical protein	-	-	-
1749	1	1277519	1277346	hypothetical protein	-	-	-
1755	1	1282628	1282311	oxidoreductase, short chain dehydrogenase/reductase family	Enoyl-(Acyl carrier protein) reductase	FabG	Dehydrogenases with different specificities (related to short-chain alcohol dehydrogenases)
1774	1	1299104	1298937	hypothetical protein	-	-	-
1813	1	1334232	1334789	ABC transporter, ATP-binding protein	ABC transporter	CcmA	ABC-type multidrug transport system, ATPase component

1813	1	1334232	1334789	ABC transporter, ATP-binding protein	Histidine kinase-, DNA gyrase B-, and HSP90-like ATPase	CcmA	ABC-type multidrug transport system, ATPase component
1849	1	1362871	1362984	hypothetical protein	-	-	-
1855	1	1367991	1368110	hypothetical protein	-	-	-
1875	1	1383218	1383102	hypothetical protein	-	-	-
1918	1	1420836	1421231	NADH dehydrogenase (EC 1.6.99.3)	DsrE/DsrF/DrsH-like family	COG2210	Peroxiredoxin family protein
1966	1	1457525	1457653	hypothetical protein	-	-	-
1	10	2723	2168	Mobile element protein	DDE domain	COG3316	Transposase and inactivated derivatives
200	12	54313	54197	hypothetical protein	-	-	-
2094	1	1555600	1555716	hypothetical protein	-	-	-
20	10	27850	27704	Retron-type RNA-directed DNA polymerase (EC 2.7.7.49)	-	-	-
2119	1	1575246	1575362	hypothetical protein	-	-	-
2122	1	1576613	1576750	hypothetical protein	-	-	-
2198	1	1640938	1641102	hypothetical protein	-	-	-
2240	1	1678913	1678800	hypothetical protein	-	-	-
2243	1	1681551	1681147	hypothetical protein	-	-	-
2288	1	1721131	1721673	hypothetical protein	-	-	-
2321	1	1754292	1754579	Branched-chain amino acid transport ATP-binding protein LivF (TC 3.A.1.4.1)	-	LivF	ABC-type branched-chain amino acid transport systems, ATPase component
2353	1	1786320	1786448	hypothetical protein	-	-	-
2382	1	1809821	1809949	hypothetical protein	-	-	-
2426	1	1847450	1847334	hypothetical protein	-	-	-
2458	1	1874247	1874489	Integral membrane protein	Protein of unknown function (DUF805)	COG3152	Predicted membrane protein

2464	1	1879598	1879810	unknown	-	-	-
2472	20	38	769	Sucrose permease, major facilitator superfamily	LacY proton/sugar symporter	-	-
2476	22	36	596	Multiple sugar ABC transporter, membrane-spanning permease protein MsmG	Binding-protein-dependent transport system inner membrane component	UgpE	ABC-type sugar transport system, permease component
2479	26	636	352	Fructokinase (EC 2.7.1.4)	pfkB family carbohydrate kinase	RbsK	Sugar kinases, ribokinase family
255	14	704	588	hypothetical protein	-	-	-
2609	2	138910	138797	2-hydroxychromene-2-carboxylate isomerase family protein	-	-	-
2612	2	141548	141682	hypothetical protein	-	-	-
2644	2	178946	179059	hypothetical protein	-	-	-
2658	2	193171	193293	hypothetical protein	-	-	-
2680	2	216957	217076	hypothetical protein	-	-	-
2685	2	221544	221657	hypothetical protein	-	-	-
2726	2	264073	264237	hypothetical protein	-	-	-
2728	2	265186	264653	hypothetical protein	Protein of unknown function (DUF3231)	-	-
2729	2	265293	265159	hypothetical protein	-	-	-
2735	2	270456	270701	PTS system, N-acetylmuramic acid-specific IIB component (EC 2.7.1.69) / PTS system, N-acetylmuramic acid-specific IIC component	phosphotransferase system, EIIB	PtsG	Phosphotransferase system IIB components

27	10	35079	35210	hypothetical protein	-	-	-
2825	2	361171	361290	hypothetical protein	-	-	-
2827	2	362378	362497	hypothetical protein	-	-	-
2884	2	411090	411248	hypothetical protein	-	-	-
28	10	35261	35389	hypothetical protein	-	-	-
2910	2	437717	437565	hypothetical protein	-	-	-
2952	2	481833	481997	hypothetical protein	Four helix bundle sensory module for signal transduction	-	-
2961	2	492560	493648	FIG01237025: hypothetical protein	Glycosyl transferase 4-like	RfaG	Glycosyltransferase
2961	2	492560	493648	FIG01237025: hypothetical protein	Glycosyl transferases group 1	RfaG	Glycosyltransferase
2962	2	494296	494427	hypothetical protein	-	-	-
2963	2	494625	495944	Membrane protein involved in the export of O-antigen, teichoic acid lipoteichoic acids	Polysaccharide biosynthesis protein	RfbX	Membrane protein involved in the export of O-antigen and teichoic acid
2964	2	495957	496874	Glycosyltransferase (EC 2.4.1.-)	Glycosyl transferase family 2	WcaA	Glycosyltransferases involved in cell wall biogenesis
2965	2	497258	498334	hypothetical protein	EpsG family	-	-
2	10	2878	2696	Mobile element protein	-	COG3316	Transposase and inactivated derivatives
3004	2	544766	544879	hypothetical protein	-	-	-
3044	2	582994	583107	hypothetical protein	-	-	-
3060	2	597423	597560	hypothetical protein	-	-	-
3098	2	637720	639020	Methylthioribulose-1-phosphate dehydratase (EC 4.2.1.109)	Class II Aldolase and Adducin N-terminal domain	AraD	Ribulose-5-phosphate 4-epimerase and related epimerases and aldolases
3098	2	637720	639020	Methylthioribulose-1-phosphate	Class II Aldolase and Adducin N-terminal domain	COG4359	Uncharacterized conserved protein

				dehydratase (EC 4.2.1.109)			
3098	2	637720	639020	Methylthioribulose-1-phosphate dehydratase (EC 4.2.1.109)	haloacid dehalogenase-like hydrolase	AraD	Ribulose-5-phosphate 4-epimerase and related epimerases and aldolases
3098	2	637720	639020	Methylthioribulose-1-phosphate dehydratase (EC 4.2.1.109)	haloacid dehalogenase-like hydrolase	COG4359	Uncharacterized conserved protein
3109	2	649872	649738	hypothetical protein	-	-	-
3122	2	662792	663028	hypothetical protein	-	-	-
3135	2	670988	671284	DnaJ-class molecular chaperone CbpA	-	-	-
3167	2	702285	702404	hypothetical protein	-	-	-
31	10	38930	38298	Transporter	Cysteine-rich secretory protein family	COG2340	Uncharacterized protein with SCP/PR1 domains
31	10	38930	38298	Transporter	Cysteine-rich secretory protein family	COG5263	FOG: Glucan-binding domain (YG repeat)
31	10	38930	38298	Transporter	Putative cell wall binding repeat	COG2340	Uncharacterized protein with SCP/PR1 domains
31	10	38930	38298	Transporter	Putative cell wall binding repeat	COG5263	FOG: Glucan-binding domain (YG repeat)
3200	2	734767	734567	hypothetical protein	-	-	-
3257	2	789835	789978	hypothetical protein	-	-	-
3262	2	791946	791527	Cell division protein FtsW	-	-	-
3356	2	887702	887827	hypothetical protein	-	-	-
3386	2	916238	915768	hypothetical protein	DinB superfamily	-	-
3394	2	924310	924429	hypothetical protein	-	-	-
33	10	41087	41248	hypothetical protein	Family of unknown function	-	-
3401	2	931011	930388	Putative threonine efflux protein	LysE type translocator	RhtB	Putative threonine efflux protein
3410	2	939368	938529	Beta-lactamase (EC 3.5.2.6)	Beta-lactamase	AmpC	Beta-lactamase class C and other penicillin binding proteins

3410	2	939368	938529	Beta-lactamase (EC 3.5.2.6)	Domain of unknown function (DUF3471)	AmpC	Beta-lactamase class C and other penicillin binding proteins
3416	2	943671	943874	hypothetical protein	-	-	-
3417	2	944057	943938	hypothetical protein	-	-	-
3419	2	945263	946876	probable protein p60 precursor	NlpC/P60 family	Spr	Cell wall-associated hydrolases (invasion-associated proteins)
3420	2	947211	947387	hypothetical protein	-	-	-
3425	2	951169	951318	hypothetical protein	-	-	-
3430	2	953820	953963	hypothetical protein	-	AraJ	Arabinose efflux permease
3435	2	957126	957401	hypothetical protein	-	-	-
3440	2	962204	962737	GCN5-related N-acetyltransferase	Acetyltransferase (GNAT) family	COG1247	Sortase and related acyltransferases
3441	2	963095	962718	Choline binding protein A	-	-	-
3475	2	1002472	1002618	hypothetical protein	-	-	-
3510	2	1040371	1041087	Formiminoglutamase (EC 3.5.3.8)	Arginase family	SpeB	Arginase/agmatinase/formimionoglutamate hydrolase, arginase family
3521	2	1054945	1055241	Transcriptional regulator, MerR family	MerR, DNA binding	SoxR	Predicted transcriptional regulators
3536	2	1064718	1064855	hypothetical protein	-	-	-
3538	2	1065740	1065862	hypothetical protein	-	-	-
3567	2	1092981	1093229	hypothetical protein	-	-	-
3569	2	1093879	1094115	hypothetical protein	-	-	-
3586	2	1109811	1109530	Spore germination protein GerKB	-	-	-
3609	2	1132923	1132723	hypothetical protein	Small Multidrug Resistance protein	EmrE	Membrane transporters of cations and cationic drugs
3611	2	1134237	1134356	hypothetical protein	-	-	-
3616	2	1138006	1138119	hypothetical protein	-	-	-
3621	2	1142986	1142696	Glucose dehydrogenase [pyrroloquinoline-quinone]	-	-	-

3622	2	1144126	1143179	Glucose dehydrogenase [pyrroloquinoline-quinone]	Glucose / Sorbosone dehydrogenase	-	-
3632	2	1150549	1150668	hypothetical protein	-	-	-
3661	2	1181189	1181368	4-carboxymuconolactone decarboxylase (EC 4.1.1.44)	Carboxymuconolactone decarboxylase family	COG0599	Uncharacterized homolog of gamma-carboxymuconolactone decarboxylase subunit
366	17	9242	8781	hypothetical protein	Protein of unknown function (DUF3231)	-	-
3676	2	1191349	1191230	hypothetical protein	-	-	-
368	17	11750	12106	FIG01249396: hypothetical protein	-	-	-
3693	3	7025	7180	hypothetical protein	-	-	-
36	10	43276	43389	hypothetical protein	-	-	-
3700	3	14159	14350	hypothetical protein	-	-	-
3715	3	31900	31769	hypothetical protein	-	-	-
3733	3	49729	49845	hypothetical protein	-	-	-
3744	3	58703	58822	hypothetical protein	-	-	-
375	17	17383	17547	hypothetical protein	-	-	-
3763	3	82549	82683	hypothetical protein	-	-	-
376	17	17887	17708	hypothetical protein	-	-	-
377	17	18354	18076	Maltose O-acetyltransferase (EC 2.3.1.79)	Bacterial transferase hexapeptide (six repeats)	WbbJ	Acetyltransferase (isoleucine patch superfamily)
37	10	43440	43565	hypothetical protein	-	-	-
3811	3	135719	135832	hypothetical protein	-	-	-
3816	3	137753	137881	Methyltransferase	-	-	-
3827	3	144985	145101	hypothetical protein	-	-	-
3830	3	146906	146754	hypothetical protein	-	-	-
394	19	1404	1553	hypothetical protein	-	-	-
395	19	1852	1721	hypothetical protein	-	-	-

3976	3	286586	286711	hypothetical protein	-	-	-
3990	3	299866	299982	hypothetical protein	-	-	-
400	19	5989	5840	hypothetical protein	-	-	-
4038	3	344851	344693	hypothetical protein	-	-	-
405	19	9818	9540	hypothetical protein	Protein of unknown function (DUF3967)	-	-
4089	3	392883	393113	hypothetical protein	Regulatory protein YrvL	-	-
4090	3	393292	393411	hypothetical protein	-	-	-
409	19	12729	12604	hypothetical protein	-	-	-
410	19	13683	13525	hypothetical protein	Family of unknown function	-	-
4159	3	467160	467639	Signal recognition particle, subunit Ffh SRP54 (TC 3.A.5.1.1)	-	-	-
4164	3	471084	471269	hypothetical protein	-	-	-
4166	3	471843	472535	Phage-associated homing endonuclease	HNH endonuclease	-	-
4167	3	472617	472781	hypothetical protein	-	-	-
4168	3	473077	472835	hypothetical protein	-	-	-
4201	3	501807	501920	hypothetical protein	-	-	-
4254	3	552541	552876	hypothetical protein	-	-	-
4261	3	559387	559506	hypothetical protein	-	-	-
4332	3	630044	629472	BH0573 unknown	-	-	-
4333	3	630192	630067	hypothetical protein	-	-	-
4374	3	667397	667516	hypothetical protein	-	-	-
4391	3	684857	684744	hypothetical protein	-	-	-
4405	3	695623	695745	hypothetical protein	-	-	-
4472	3	752029	751880	hypothetical protein	-	-	-
4473	3	752382	752188	hypothetical protein	-	-	-
4485	3	764091	763891	hypothetical protein	-	-	-
4507	3	784800	784922	hypothetical protein	-	-	-

4562	3	837207	837040	hypothetical protein	Protein of unknown function, DUF600	-	-
4563	3	837462	837244	Repetitive hypothetical protein near ESAT cluster, SA0282 homolog	Protein of unknown function, DUF600	-	-
4564	3	837641	837468	Putative toxin component near putative ESAT-related proteins, repetitive / Repetitive hypothetical protein near ESAT cluster, SA0282 homolog	DNA/RNA non-specific endonuclease	-	-
4566	3	839425	838493	Putative toxin component near putative ESAT-related proteins, repetitive / Repetitive hypothetical protein near ESAT cluster, SA0282 homolog	A nuclease family of the HNH/ENDO VII superfamily with conserved AHH	-	-
4568	3	841399	840470	Lmo0066 homolog within ESAT-6 gene cluster, similarity to ADP-ribosylating toxins	The BURPS668_1122 family of deaminases	-	-
461	1	49775	49897	hypothetical protein	-	-	-
4621	3	894952	894815	hypothetical protein	-	-	-
4631	3	902503	902177	Syd protein	Syd protein (SUKH-2)	-	-
4632	3	902696	902553	Syd protein	-	-	-
4645	3	918360	918112	hypothetical protein	-	-	-
4646	3	918572	918402	hypothetical protein	Immunity protein 50	-	-
4714	4	35421	35534	hypothetical protein	-	-	-

4772	4	87567	87229	Transporter	Protein of unknown function (DUF502)	COG2928	Uncharacterized conserved protein
4891	4	208995	209120	hypothetical protein	-	-	-
4991	4	308914	308597	FIG00672752: hypothetical protein	Putative threonine/serine exporter	COG2966	Uncharacterized conserved protein
4992	4	309273	308887	FIG00672752: hypothetical protein	Putative threonine/serine exporter	COG2966	Uncharacterized conserved protein
5004	4	322729	322613	hypothetical protein	-	-	-
5083	5	45080	45283	L-alanoyl-D-glutamate peptidase	-	-	-
5084	5	45443	45601	L-alanoyl-D-glutamate peptidase	D-alanyl-D-alanine carboxypeptidase	-	-
5143	5	108492	108659	hypothetical protein	-	-	-
5159	5	120670	120789	hypothetical protein	-	-	-
516	1	105631	105509	hypothetical protein	-	-	-
5199	6	1907	2032	hypothetical protein	-	-	-
5200	6	2810	2619	hypothetical protein	Family of unknown function	-	-
5201	6	4025	3774	hypothetical protein	-	-	-
5208	6	10562	10050	FIG01238583: hypothetical protein	Protein of unknown function (DUF3231)	-	-
5209	6	10846	10730	hypothetical protein	-	-	-
5211	6	11610	12287	hypothetical protein	PAP2 superfamily	PgpB	Membrane-associated phospholipid phosphatase
5214	6	15859	16893	RsbR, positive regulator of sigma-B	STAS domain	SpoIIAA	Anti-anti-sigma regulatory factor (antagonist of anti-sigma factor)
5215	6	18260	17406	hypothetical protein	-	-	-
5218	6	20157	20321	hypothetical protein	-	-	-
5220	6	22805	22933	hypothetical protein	-	-	-
5226	6	29395	29195	RsbR, positive regulator of sigma-B	-	-	-
5230	6	33816	33694	hypothetical protein	-	-	-

5231	6	35317	35577	RsbR, positive regulator of sigma-B	-	-	-
5235	6	39249	39115	hypothetical protein	Poly-gamma-glutamate hydrolase	-	-
5236	6	39812	39672	hypothetical protein	-	-	-
5247	6	51397	51546	hypothetical protein	-	-	-
5252	6	56151	56032	hypothetical protein	-	-	-
5278	6	77867	77989	hypothetical protein	-	-	-
5279	6	78518	78649	hypothetical protein	-	-	-
527	1	115589	115702	hypothetical protein	-	-	-
5286	6	85468	85602	hypothetical protein	-	-	-
5291	6	88313	88104	hypothetical protein	YolD-like protein	-	-
5304	6	102547	104076	hypothetical protein	PIN domain	-	-
5305	6	104368	104186	hypothetical protein	NA	-	-
5306	6	105420	104584	hypothetical protein	Predicted pPIWI-associating nuclease	-	-
5307	6	107695	105512	hypothetical protein	SEC-C motif	COG3318	Predicted metal-binding protein related to the C-terminal domain of SecA
5308	6	107969	107835	hypothetical protein	-	-	-
5312	6	112753	113109	Na+/H+ antiporter	Sodium/hydrogen exchanger family	KefB	Kef-type K+ transport systems, membrane components
5316	6	117225	118187	Sporulation control protein Spo0M	SpoOM protein	Spo0M	Sporulation control protein
5317	6	118646	119029	Transcriptional regulator, MerR family	MerR HTH family regulatory protein	SoxR	Predicted transcriptional regulators
5318	6	119050	119742	probable metal-dependent peptidase	Putative neutral zinc metallopeptidase	COG2738	Predicted Zn-dependent protease
5320	6	121413	121243	hypothetical protein	-	-	-
5324	6	125584	125456	hypothetical protein	-	-	-
5329	6	130949	131539	Choline binding protein A	L,D-transpeptidase catalytic domain	ErfK	Uncharacterized protein conserved in bacteria

5336	6	138719	138585	hypothetical protein	Glyoxalase/Bleomycin resistance protein/Dioxygenase superfamily	-	-
5341	6	145333	144587	Retron-type RNA-directed DNA polymerase (EC 2.7.7.49)	Group II intron, maturase-specific domain	-	-
5342	6	146693	145449	Retron-type RNA-directed DNA polymerase (EC 2.7.7.49)	Group II intron, maturase-specific domain	COG3344	Retron-type reverse transcriptase
5342	6	146693	145449	Retron-type RNA-directed DNA polymerase (EC 2.7.7.49)	Reverse transcriptase (RNA-dependent DNA polymerase)	COG3344	Retron-type reverse transcriptase
5449	7	100023	99886	hypothetical protein	-	-	-
5459	7	109617	109270	hypothetical protein	-	-	-
5489	8	239	78	hypothetical protein	-	-	-
5490	8	346	564	cell wall lytic activity	NlpC/P60 family	Spr	Cell wall-associated hydrolases (invasion-associated proteins)
5494	8	4982	4869	hypothetical protein	-	-	-
5495	8	6681	6226	Cytochrome c oxidase (B(O/a)3-type) chain II (EC 1.9.3.1)	Cupredoxin-like domain	CyoA	Heme/copper-type cytochrome/quinol oxidases, subunit 2
5496	8	7003	8253	Putative type II restriction enzyme NmeDIP (EC 3.1.21.4) (Endonuclease NmeDIP) (R.NmeDIP)	-	-	-
54	10	57856	57485	CDS_ID OB0576	Protein of unknown function (DUF2642)	-	-
5504	8	17945	18085	hypothetical protein	-	-	-

5505	8	19058	19462	Transcriptional regulator, MecI family	Penicillinase repressor	COG3682	Predicted transcriptional regulator
5506	8	19462	20316	Peptidase M48, Ste24p precursor	BlaR1 peptidase M56	HtpX	Zn-dependent protease with chaperone function
5507	8	20434	20673	hypothetical protein	-	-	-
5518	8	29236	28748	GCN5-related N-acetyltransferase	Acetyltransferase (GNAT) domain	COG1247	Sortase and related acyltransferases
5519	8	29596	29907	Arsenical resistance operon repressor	Bacterial regulatory protein, arsR family	ArsR	Predicted transcriptional regulators
5521	8	32539	30671	Phage protein	HNH endonuclease	McrA	Restriction endonuclease
5527	8	37545	37414	hypothetical protein	-	-	-
5528	8	37965	39218	transcriptional regulator, MerR family	Helix-turn-helix	HipB	Predicted transcriptional regulators
5528	8	37965	39218	transcriptional regulator, MerR family	Helix-turn-helix	PilF	Tfp pilus assembly protein PilF
5528	8	37965	39218	transcriptional regulator, MerR family	Tetratricopeptide repeat	HipB	Predicted transcriptional regulators
5528	8	37965	39218	transcriptional regulator, MerR family	Tetratricopeptide repeat	PilF	Tfp pilus assembly protein PilF
5529	8	39523	39233	Arsenical resistance operon repressor	Helix-turn-helix domain	ArsR	Predicted transcriptional regulators
5531	8	41054	40761	hypothetical protein	-	-	-
5533	8	41797	42699	FIG01237413: hypothetical protein	Predicted permease	COG0701	Predicted permeases
5534	8	42715	42849	hypothetical protein	-	-	-
5535	8	43907	43758	hypothetical protein	-	-	-
5538	8	45699	45824	hypothetical protein	-	-	-
5540	8	47224	48378	hypothetical protein	-	-	-
5541	8	48431	48646	hypothetical protein	-	-	-
5542	8	48639	49349	hypothetical protein	-	-	-

5548	8	57527	58255	Organomercurial lyase (EC 4.99.1.2)	Alkylmercury lyase	-	-
5551	8	59200	59313	hypothetical protein	-	-	-
5552	8	59434	59700	hypothetical protein	-	CopZ	Copper chaperone
5554	8	62388	62077	hypothetical protein	-	-	-
5555	8	62697	62413	hypothetical protein	-	-	-
5593	8	96382	96582	hypothetical protein	-	-	-
5595	8	98654	98490	hypothetical protein	-	-	-
5596	8	99282	99163	hypothetical protein	-	-	-
5607	8	114354	115532	Stage II sporulation protein P	Stage II sporulation protein P (SpoIIP)	-	-
5611	8	118759	118923	hypothetical protein	NA	-	-
5612	8	118926	119255	Putative 2Fe-2S ferredoxin CbiW involved in B12 biosynthesis	Thioredoxin-like [2Fe-2S] ferredoxin	COG3411	Ferredoxin
5614	8	120129	121547	Osmosensitive K+ channel histidine kinase KdpD (EC 2.7.3.-)	HAMP domain	BaeS	Signal transduction histidine kinase
5614	8	120129	121547	Osmosensitive K+ channel histidine kinase KdpD (EC 2.7.3.-)	His Kinase A (phospho-acceptor) domain	BaeS	Signal transduction histidine kinase
5614	8	120129	121547	Osmosensitive K+ channel histidine kinase KdpD (EC 2.7.3.-)	Histidine kinase-, DNA gyrase B-, and HSP90-like ATPase	BaeS	Signal transduction histidine kinase
5625	9	6515	6378	hypothetical protein	Sigma-70, region 4	RpoE	DNA-directed RNA polymerase specialized sigma subunit, sigma24 homolog
5626	9	6814	6629	hypothetical protein	Sigma-70 region 2	RpoE	DNA-directed RNA polymerase specialized sigma subunit, sigma24 homolog

5646	9	27827	28822	Alcohol dehydrogenase (EC 1.1.1.1)	Alcohol dehydrogenase GroES-like domain	Qor	NADPH:quinone reductase and related Zn-dependent oxidoreductases
5646	9	27827	28822	Alcohol dehydrogenase (EC 1.1.1.1)	Zinc-binding dehydrogenase	Qor	NADPH:quinone reductase and related Zn-dependent oxidoreductases
5653	9	34857	35015	hypothetical protein	-	-	-
5661	9	43582	42740	Hydrolase, alpha/beta fold family	alpha/beta hydrolase fold	MhpC	Predicted hydrolases or acyltransferases (alpha/beta hydrolase superfamily)
5670	9	49967	50080	hypothetical protein	-	-	-
5677	9	55301	55465	hypothetical protein	-	-	-
5679	9	58052	58273	Flavohemoprotein (Hemoglobin-like protein) (Flavohemoglobin) (Nitric oxide dioxygenase) (EC 1.14.12.17)	-	Hmp	Flavodoxin reductases (ferredoxin-NADPH reductases) family 1
5681	9	59834	59715	hypothetical protein	-	-	-
5682	9	60268	60068	hypothetical protein	-	-	-
5691	9	70437	69715	hypothetical protein	-	-	-
5692	9	70686	70558	hypothetical protein	-	-	-
56	10	60437	59139	UDP-glucose dehydrogenase (EC 1.1.1.22)	UDP-glucose/GDP-mannose dehydrogenase family, central domain	WecC	UDP-N-acetyl-D-mannosaminuronate dehydrogenase
56	10	60437	59139	UDP-glucose dehydrogenase (EC 1.1.1.22)	UDP-glucose/GDP-mannose dehydrogenase family, NAD binding domain	WecC	UDP-N-acetyl-D-mannosaminuronate dehydrogenase
56	10	60437	59139	UDP-glucose dehydrogenase (EC 1.1.1.22)	UDP-glucose/GDP-mannose dehydrogenase family, UDP binding domain	WecC	UDP-N-acetyl-D-mannosaminuronate dehydrogenase
5700	9	79955	80116	hypothetical protein	-	-	-
5705	9	83651	83319	hypothetical protein	YolD-like protein	-	-
5706	9	83835	83665	DNA polymerase IV (EC 2.7.7.7)	-	-	-

5713	9	88102	88224	hypothetical protein	-	-	-
5717	9	92171	90987	Translation elongation factor Tu	Elongation factor Tu C-terminal domain	TufB	GTPases - translation elongation factors
5717	9	92171	90987	Translation elongation factor Tu	Elongation factor Tu domain 2	TufB	GTPases - translation elongation factors
5717	9	92171	90987	Translation elongation factor Tu	Elongation factor Tu GTP binding domain	TufB	GTPases - translation elongation factors
5724	9	97008	95590	Lead, cadmium, zinc and mercury transporting ATPase (EC 3.6.3.3) (EC 3.6.3.5); Copper-translocating P-type ATPase (EC 3.6.3.4)	E1-E2 ATPase	ZntA	Cation transport ATPase
5724	9	97008	95590	Lead, cadmium, zinc and mercury transporting ATPase (EC 3.6.3.3) (EC 3.6.3.5); Copper-translocating P-type ATPase (EC 3.6.3.4)	haloacid dehalogenase-like hydrolase	ZntA	Cation transport ATPase
5730	9	102637	102789	hypothetical protein	-	-	-
5735	9	105364	105248	hypothetical protein	-	-	-
5737	9	107188	107562	Choline binding protein A	L,D-transpeptidase catalytic domain	ErfK	Uncharacterized protein conserved in bacteria
5741	9	113259	112981	Maltose O-acetyltransferase (EC 2.3.1.79)	Bacterial transferase hexapeptide (six repeats)	WbbJ	Acetyltransferase (isoleucine patch superfamily)
593	1	184646	184527	hypothetical protein	-	-	-

59	10	64360	63110	Glycosyl transferase (EC 2.4.1.-)	Glycosyl transferases group 1	RfaG	Glycosyltransferase
59	10	64360	63110	Glycosyl transferase (EC 2.4.1.-)	Glycosyltransferase Family 4	RfaG	Glycosyltransferase
60	10	65727	64429	hypothetical protein	D-glucuronyl C5-epimerase C-terminus	-	-
61	10	66635	65958	Acetyltransferase (EC 2.3.1.-)	Bacterial transferase hexapeptide (six repeats)	GlmU	N-acetylglucosamine-1-phosphate uridyltransferase (contains nucleotidyltransferase and I-patch acetyltransferase domains)
62	10	67576	66656	hypothetical protein	Glycosyl transferases group 1	-	-
63	10	67734	68711	probable oxidoreductase	Oxidoreductase family, NAD-binding Rossmann fold	MviM	Predicted dehydrogenases and related proteins
645	1	238894	239010	hypothetical protein	-	-	-
66	10	72163	72867	hypothetical protein	-	-	-
70	10	77349	77227	hypothetical protein	-	-	-
72	10	79060	79173	hypothetical protein	-	-	-
73	10	79611	79495	hypothetical protein	-	-	-
74	10	81010	81123	hypothetical protein	Family of unknown function	-	-
75	10	81281	81484	hypothetical protein	Protein of unknown function DUF86	COG2445	Uncharacterized conserved protein
798	1	386050	386406	hypothetical protein	Domain of unknown function (DUF4912)	COG3330	Uncharacterized protein conserved in bacteria
84	11	5288	5413	hypothetical protein	-	-	-
85	11	6720	6508	hypothetical protein	-	-	-
87	11	10126	9437	Methionine ABC transporter ATP-binding protein	ABC transporter	SalX	ABC-type antimicrobial peptide transport system, ATPase component
887	1	472542	472670	hypothetical protein	-	-	-
91	11	13840	14121	hypothetical protein	Bacteriocin class IIc cyclic gassericin A-like	-	-

Table S3. Antibiotic susceptibility of the *B. megaterium* HgT21 strain.

**Antibiotic**	**Concentration (μg)**	
Ampicillin	10	Resistant
Levofloxacin	5	Susceptible
Cephalothin	30	Susceptible
Cefotaxime	30	Resistant
Chloramphenicol	30	Susceptible
Netilmicin	30	Susceptible
Cefepime	30	Susceptible
Trimethoprim / Sulfametoxazole	25	Susceptible
Ceftazidime	30	Resistant
Cefuroxime	30	Resistant
Dicloxacillin	1	Resistant
Pefloxacine	5	Susceptible
Penicillin	10*	Resistant
Tetracycline	30	Susceptible
Amikacin	30	Susceptible
Gentamicin	30	Susceptible
Ciprofloxacin	5	Susceptible
Vancomycin	30	Susceptible
Erythromycin	15	Susceptible
Ceftriaxone	30	Susceptible
Nitrofurantoin	300	Susceptible

*(U) units
(BBL Antibiotic SensiDiscs, BD. Gram Positive and Gram Negative II Multidisc, BIO RAD).
